# Fasting triglyceride concentrations are associated with markers of lipid metabolism and glucose homeostasis in healthy, non-obese dogs in lean and overweight condition

**DOI:** 10.3389/fvets.2024.1406322

**Published:** 2024-09-04

**Authors:** Carlos Gomez-Fernandez-Blanco, Dominique Peeters, Frédéric Farnir, Katja Höglund, Vassiliki Gouni, Maria Wiberg, Jakob Lundgren Willesen, Sofia Hanås, Kathleen McEntee, Laurent Tiret, Jens Häggström, Hannes Lohi, Valérie Chetboul, Merete Fredholm, Eija Seppälä, Anne-Sophie Lequarré, Alexander James German, Anne-Christine Merveille

**Affiliations:** ^1^Department of Clinical Sciences, College of Veterinary Medicine, University of Liège, Liège, Belgium; ^2^Department of Sustainable Animal Resources, FARAH, College of Veterinary Medicine, University of Liège, Liège, Belgium; ^3^Department of Animal Biosciences, Swedish University of Agricultural Sciences, Uppsala, Sweden; ^4^INSERM, IMRB, Université Paris-Est Créteil, Créteil, France; ^5^IMRB, École Nationale Vétérinaire d’Alfort, Maisons-Alfort, France; ^6^Department of Equine and Small Animal Medicine, Faculty of Veterinary Medicine, University of Helsinki, Helsinki, Finland; ^7^Department of Veterinary Clinical Sciences, University of Copenhagen, Frederiksberg, Denmark; ^8^Department of Clinical Sciences, Swedish University of Agricultural Sciences, Uppsala, Sweden; ^9^Evidensia Specialist Animal Hospital Strömsholm, Strömsholm, Sweden; ^10^Faculty of Medicine, Université Libre de Bruxelles (ULB), Brussels, Belgium; ^11^Department of Medical and Clinical Genetics, University of Helsinki, Helsinki, Finland; ^12^Department of Veterinary Biosciences, University of Helsinki, Helsinki, Finland; ^13^Folkhälsan Research Center, Helsinki, Finland; ^14^Department of Veterinary and Animal Sciences, University of Copenhagen, Frederiksberg, Denmark; ^15^Institute of Life Course and Medical Sciences, University of Liverpool, Liverpool, United Kingdom

**Keywords:** canine, metabolic syndrome, BCS, triglycerides, insulin, fructosamine, cholesterol, free fatty acids

## Abstract

Serum triglyceride concentrations increase in dogs with obesity, which is typically assessed by body condition score (BCS), however little is known about changes that take place in non-obese dogs in overweight condition. Further, the associations of triglyceride levels with other markers of energy homeostasis are poorly characterised in healthy animals. The present study aimed to evaluate associations between both BCS and triglyceride concentrations with other markers of lipid and glucose metabolism in healthy, non-obese dogs, as well as to assess whether these markers change significantly in non-obese dogs with overweight as compared to their lean counterparts. Serum concentrations of cholesterol, free fatty acids, triglycerides, insulin, glucose and fructosamine were measured in 532 healthy, client-owned dogs, assigned either to ‘lean’ (BCS: 3–5) or ‘overweight’ (BCS: 6–7) categories. Generalised linear mixed models were used to assess associations between BCS categories, triglyceride concentrations and other variables, correcting for the effect of breed. Compared with lean dogs, overweight dogs had a greater serum cholesterol concentration (95% CI, 5.3–6.2 mmol/L or 205–237 mg/dL versus 5.1–5.4 mmol/L or 198–210 mg/dL, *p* = 0.0032), insulin concentration (95% CI, 17.5–22.1 μU/ml versus 16.7–18.0 μU/ml, *p* = 0.0374) and were older (95% CI, 4.0–5.3 versus 3.4–3.7 years, *p* = 0.0005). Triglyceride concentrations were positively associated with fructosamine (*r*^2^ = 0.31, *p* = 0.0012), cholesterol (*r*^2^ = 0.25, *p* < 0.0001), insulin (*r*^2^ = 0.14, *p* = 0.0030) and glucose (*r*^2^ = 0.10, *p* = 0.0014) concentrations, and negatively associated with free fatty acid concentrations (*r*^2^ = 0.11, *p* < 0.0001). However, there was no association between triglyceride concentrations and age. In conclusion, both BCS and triglyceride concentrations were associated with other markers of glucose and lipid metabolism in non-obese healthy dogs, amongst which those with overweight showed metabolic changes as compared to their lean counterparts. Triglyceride concentrations were associated with an increase in insulin and fructosamine concentrations that might reflect an early-phase impairment in glucose tolerance which, surprisingly, was concurrent with lower basal free fatty acid concentrations.

## Introduction

Metabolic syndrome (MS) is an entity comprising multiple cardiovascular and metabolic risk factors in humans, characterised by a state of chronic, subclinical inflammation (1). Features of human MS include combinations of increased visceral fat (abdominal obesity), systemic hypertension, increased circulating triglyceride (TG) concentrations, decreased high-density lipoprotein (HDL) cholesterol concentration, and increased fasting glucose concentration (suggesting insulin resistance) (2). Human beings with MS have a greater risk of developing type 2 diabetes mellitus (DM) and cardiovascular complications (2).

Like humans, and as opposed to traditional animal models of obesity (e.g., rodents), obesity develops spontaneously in dogs. For this reason, canine models of obesity are gaining interest, as they may provide insights into the mechanisms of disease and potential new therapeutic approaches (3).

Dogs also suffer from obesity-related metabolic dysfunction (ORMD), but the term metabolic syndrome is avoided because, although some components are shared (e.g., hypercholesterolaemia, hypertriglyceridaemia and insulin resistance), dogs do not develop the obesity-related diseases of human MS, such as atherosclerosis, stroke or type 2 DM (4–7). Studies on canine ORMD have mostly examined dogs with manifest obesity, whilst metabolic changes of dogs in a milder overweight condition have been less well characterised, despite the known association of this condition with comorbidities in the canine species (8–11).

Some people with obesity remain metabolically healthy, at least in the short term (12), during which time their risk of comorbidities is less, they do not develop insulin resistance; are normotensive; and the concentrations of glucose, TG, HDL cholesterol and high-sensitivity C-reactive protein (hsCRP) are all within reference limits (13). Conversely, some normal-weight individuals develop the characteristics of MS despite having a normal body fat mass (14). These ‘metabolically-unhealthy normal-weight’ people can be identified by a thorough biochemical assessment, where increased TG and C-reactive protein (CRP) concentrations, as well as decreased HDL-cholesterol and adiponectin concentrations will be identified (14). Similarly, different biochemical phenotypes have been described in canine obesity; for example, adiponectin concentration was less and plasma insulin concentration greater in dogs with obesity that met the criteria of ORMD, which was defined as having obesity plus two other criteria amongst: TG >200 mg/dL (>2.26 mmol/L), total cholesterol >300 mg/dL (>7.8 mmol/L), systolic blood pressure > 160 mmHg, and either a fasting plasma glucose >100 mg/dL (>5.6 mmol/L) or previously diagnosed diabetes mellitus (15, 16). In these studies, the presence of obesity was defined using a 9-unit BCS (body condition score), a validated semi-quantitative system using assessment and palpation that correlates well with body fat mass determined by dual-energy X-ray absorptiometry (8, 17–19), making it suitable for clinical use as well as in studies on overweight and obesity in dogs. Although descriptions in some BCS categories do mention muscle mass (e.g., “obvious loss of muscle mass” in BCS 1), the system mainly assesses subcutaneous and abdominal adipose tissue.

The first aim of this study was to compare various metabolic variables associated with glucose and fat metabolism in a large cohort of non-obese dogs that were either lean or in overweight condition, as defined by BCS. A second aim was to investigate associations between TG concentrations and the same metabolic variables. We hypothesise that non-obese overweight dogs will show biochemical evidence of metabolic changes as compared to lean individuals, and that TG levels as a sole marker will also be associated to metabolic changes in a general population of non-obese dogs.

## Materials and methods

A canine database (European LUPA project) used to study genetic determinants of disease (20) was retrieved. Five centres had participated in the original study between 2009 and 2010: University of Liège (Belgium), University of Copenhagen (Denmark), Swedish University of Agricultural Sciewnces (Sweden), University of Helsinki (Finland) and the National Veterinary School of Maisons-Alfort (France). All centres used the same standardised protocols for recruitment and characterisation of dogs. This is therefore a prospective study which uses an already existing database with no modifications in the original protocol. No retrospective data were generated for the purpose of this study.

Client-owned, pure-bred dogs were recruited, and included different breed cohorts in order to represent a wide range of various phenotypic features. Dogs were 1–7 years old and were genetically unrelated. To minimise potential effects of the oestrous cycle on metabolic parameters, each breed cohort comprised a single sex, namely intact males or female dogs that were spayed or in anoestrus (checked by a vaginal smear). Health status was checked by reviewing history, physical examination, laboratory analyses (including routine haematology and serum biochemistry), and with a thorough cardiovascular investigation comprising ECG recording and echocardiographic examination. After visual assessment and palpation, each dog was assigned a BCS category using the one-to-nine point scale (17); “lean” (including dogs with ideal BCS – 4 to 5 – and slightly thin dogs – BCS 3) and “overweight” (BCS 6–7). Dogs were excluded if they showed clinical signs of any disease (in order to identify changes related to fat accumulation alone), were markedly underweight (BCS ≤2) or had obesity [BCS 8–9, as defined by Brooks et al. (21)].

Three weeks before the study, owners were asked to feed their dogs exclusively with a commercial dry food diet of their choice, avoiding any treats or table food. Dogs were fasted for at least 12 h before blood sampling and, after collection, blood was centrifuged within 30 min and serum then aliquoted. In most centres, serum aliquots were immediately frozen at −80°C; in one centre, samples were frozen at −20°C for the first 2 weeks after collection, before being transferred to a − 80°C thereafter. All samples were subsequently sent to the same laboratory for analysis.

The analytes selected for the present study were chosen based on their reported association with obesity in humans and dogs (2, 4, 15, 22–44). These included markers of lipid metabolism (cholesterol, TG and free fatty acids, FFA), glucose homeostasis (glucose, fructosamine, and insulin) and inflammation (CRP). Insulin was determined by radioimmunoassay (DiaSorin S.p.A Italy) and all other variables were determined by spectrophotometry using a clinical chemistry analyzer (Konelab 60i, Thermo Electron Co, Finland). Thermo Electron kits were used for glucose, CRP, cholesterol, and TG. Kits for fructosamine and FFA were provided by Horiba ABX (Montpellier, France) and Wako Chemical Gmbh (Neuss, Germany), respectively.

To determine assay variability, duplicate samples from 10 dogs randomly selected from the cohort were submitted to the same laboratory 3 months after the first analyses; coefficients of variation were 6% for insulin and ≤ 5% for FFA, cholesterol, TG, glucose and fructosamine. Outliers were inspected with the Reference Value Advisor (45) using the Tukey method (46). All outliers were used in the statistical analyses, since they were considered compatible with physiological values.

All statistical analyses were performed using an open-access statistical language and environment (RStudio 2023.03.0 Build 386). Generalised linear mixed models were performed with the function lm() of the lme4 package (47). For all analyses in this study, *p*-values <0.05 were considered to be statistically significant, and a Bonferroni correction was applied whenever multiple comparisons were made.

Differences in concentrations of all biochemical variables between lean and overweight dogs were investigated with a generalised linear mixed model, which included BCS and breed, as well as their interaction. A second generalised linear mixed model was created to examine associations between concentrations of TG and other biochemical variables; this second model included TG and breed, as well as their interaction. Type III sums of squares were evaluated. For both models, whenever the interaction term with breed was significant (i.e., BCS and breed for model 1; TG and breed for model 2), the association between the explanatory variable (BCS or TG) and the outcome variable was tested within each breed using non-parametric methods (Mann–Whitney or Spearman’s correlation method, respectively). All models were validated by checking the normal distribution (evaluated by visual inspection of histograms and the Shapiro–Wilk test) and homoscedasticity (evaluated with the Breusch-Pagan test) of residuals. If the assumptions were not met, non-parametric equivalent tests were used instead (e.g., Spearman’s Rank correlation or Mann–Whitney test as appropriate).

### Ethics statement

This study was performed following the recommendations of the Guide for the Care and Use of Laboratory Animals of the National Institutes of Health (48), and was approved by the Ethical Committees of the LUPA project (20). Written consent of all owners was obtained.

## Results

In total, 532 dogs met the inclusion criteria, with 9 different breeds represented: Boxer (15 dogs), Belgian Shepherd Dog (BSD, 124 dogs), Cavalier King Charles Spaniel (CKCS, 35 dogs), Dachshund (40 dogs), Doberman (39 dogs), Finnish Lapphund (45 dogs), German Shepherd Dog (GSD, 65 dogs), Labrador Retriever (125 dogs), and Newfoundland (44 dogs). All cohorts comprised only male dogs, except for the Newfoundland and Labrador retriever cohorts that comprised only females and dogs of both sexes (73 females and 52 males), respectively. Distribution of dogs by centre, breed, and sex is shown in Table 1; some breeds were unique to one centre whilst others were distributed amongst centres.

**Table 1:** Distribution of dogs by centre, breed, and sex.

	Belgium	Denmark	Finland	France	Sweden	Total
BSD	97M			28M		124
Boxer					15M	15
CKCS					35M	35
Dachshund			24M		16M	40
Doberman				39M		39
Finnish Lapphund			45M			45
GSD	17M		49M			65
Labrador retriever	7M	44F		29F	45M	125
Newfoundland		44F				44
Total	121	88	118	96	111	532

M, Male; F, Female; BSD, Belgian Shepherd Dog; CKCS, Cavalier King Charles Spaniel; GSD, German Shepherd Dog.

There were 120 dogs in the “lean” category (median BCS = 5; IQR =1; mean BCS = 4.3; range, 2–5) and 407 “overweight” dogs (median BCS = 6; IQR = 0; mean BCS = 6.1; range, 6–7). Table 2 shows the distribution of BCS amongst breeds. Median BCS was 3 in Finnish Lapphunds (range: 3–4); 4 in GSDs (range: 3–4), Dachshunds (range: 3–5), BSDs (range: 4–5) and Boxers (range: 4–5); 5 in Dobermans (range: 5–5), CKCSs (range: 5–6) and Newfoundlands (range: 5–6); and 5.5 in Labrador retrievers (range: 5–6). BCS information was missing in 3 GSD and in 2 Newfoundlands.

**Table 2:** Number of dogs in each BCS, distributed by breed.

	BCS = 3	BCS = 4	BCS = 5	BCS = 6	BCS = 7
Boxer	–	8	4	3	–
BSD	22	45	51	5	1
CKCS	–	-	20	15	–
Dachshund	11	12	11	6	–
Doberman	–	2	33	4	–
Finnish Lapphund	24	12	6	2	1
GSD	22	35	4	1	–
Labrador retriever	1	8	53	59	4
Newfoundland	–	–	23	15	4
Total	80	122	205	110	10

BCS, Body Condition Score; BSD, Belgian Shepherd Dog; CKCS, Cavalier King Charles Spaniel; GSD, German Shepherd Dog.

None of the dogs in the present study met the criteria for ORMD [as defined by Tvarijonaviciute et al. (15)].

**Figure 1:**
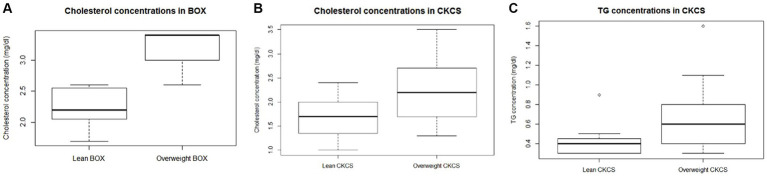
Box plots showing serum cholesterol **(A,B)** and triglyceride **(C)** concentrations in Boxer (BOX) and Cavalier King Charles Spaniel (CKCS). Bonferroni-corrected *p*-value of 0.0056. The lower, middle and upper line of each box represent the 25th percentile (bottom quartile), 50th percentile (median) and the 75th percentile (top quartile). The whiskers, where present, represent the minimum and maximum. Outliers, represented by open circles, were included in the analyses.

There was no effect of age on any analyte tested (cholesterol, *p* = 0.7424; FFA, *p* = 0.9631; TG, *p* = 0.5374; CRP, *p* = 0.3936; insulin, *p* = 0.1058; glucose, *p* = 0.6525.; fructosamine, *p* = 0.1733). After normalisation for the effect of the breed, dogs in the overweight category were older (*p* = 0.0005) and had greater serum insulin (*p* = 0.0374) and cholesterol (*p* = 0.0032) concentrations than lean dogs. An interaction between BCS category and breed was also identified for both TG and cholesterol concentrations (Table 3); cholesterol concentration was greater in overweight boxers (*p* = 0.0197) and CKCSs (*p* = 0.0053) compared with their lean counterparts; overweight CKCSs also had greater TG concentrations than lean CKCSs (*p* = 0.0023). These differences are illustrated in Figure 1.

**Table 3:** Results of a generalised linear mixed model examining the effect of BCS category, and its interaction with the effect of the breed, on age (years) and on concentrations of insulin, fructosamine, glucose, cholesterol, free fatty acids, and triglycerides in overweight and lean dogs.

Variable	Group		*P*-value^a^		
	Lean	Overweight	BCS	Breed	BCS * breed^b^
Age (years)	3.5 (3.4–3.7)	4.4 (3.9–4.9)	**0.0005**	0.0506	0.3405
Insulin			**0.0374**	**<0.0001**	0.1289
(pmol/l)	120.1 (113.2–125.7)	131.3 (122.2–141.7)			
(μU/ml)	17.3 (16.7–18.1)	18.9 (17.6–20.4)			
Fructosamine (μmol/l)	287.1 (283.6–290.6)	289.7 (283.2–296.2)	0.4820	**<0.0001**	0.4560
Glucose			0.3828	**<0.0001**	0.6783
(mmol/l)	0.05, (0.05–0.05)	0.05 (0.05–0.06)			
(mg/dl)	0.98 (0.97–0.99)	0.99 (0.97–1.01)			
Cholesterol			**0.0032**	**<0.0001**	**0.0006**
(mmol/l)	5.3 (5.1–5.4)	5.7 (5.3–6.2)			
(mg/dl)	204 (198–210)	221 (205–237)			
FFA (mEq/l)	0.87 (0.83–0.91)	0.86 (0.78–0.93)	0.7657	**0.0001**	0.7529
Triglycerides			0.9716	**<0.0001**	**0.0019**
(mmol/l)	0.005 (0.005–0.005)	0.005 (0.005–0.006)			
(mg/dl)	0.43 (0.42–0.45)	0.45 (0.40–0.51)			

Data are presented as least-squares means and 95% confidence intervals. FFA, free fatty acids. ^a^*p*-values in bold are significant at *p* < 0.05. ^b^*p*-value for effect of the interaction between BCS category and breed on the outcome variable.

Results of the model assessing associations between TG concentrations and the different outcome variables are shown in Table 4. After controlling for the effect of the breed, TG concentrations were positively associated with fructosamine (*p* = 0.001), cholesterol (*p* < 0.001), insulin (*p* = 0.003) and glucose (*p* = 0.001) concentrations, and negatively associated with FFA concentrations (*p* < 0.0001). For insulin concentration, there was also a significant interaction between TG and breed (*p* = 0.0101), and intra-breed analyses revealed positive association between TG and insulin concentrations in several breeds (Table 5).

**Table 4:** Association between triglyceride concentration and insulin, fructosamine, glucose, cholesterol, and free fatty acids.

Variable	*R*^2^	*p*-value^a^		
		Triglycerides	Breed	TG * breed^b^
Insulin	0.16	**0.0030**	**0.0257**	**0.0101**
Fructosamine	0.31	**0.0012**	**<0.0001**	0.0711
Glucose	0.10	**0.0014**	**<0.0001**	0.7750
Cholesterol	0.25	**<0.0001**	**<0.0001**	0.1572
FFA	0.10	**<0.0001**	**<0.0001**	0.0645

The *r*^2^ corresponds to the predictive percentage attributed to the whole linear model, which included the main effects of both triglycerides and breed (plus their interaction, when significant) as explanatory variables. FFA, free fatty acids. ^a^*p*-values in bold are significant at *p* < 0.05. ^b^*p*-value for effect of the interaction between triglyceride concentrations and breed on the outcome variable.

**Table 5:** Associations between triglyceride concentrations (mg/dl) and insulin (μU/ml) within individual breeds.

Breed	Spearman’s *R*^1^	*p*-value
Boxer	0.67	0.0066
BSD	0.30	0.0008
CKCS	0.11	0.5361
Dachshund	0.28	0.0819
Doberman pinshcer	0.39	0.0515
Finnish Lapphund	−0.07	0.6246
GSD	0.16	0.1973
Labrador retriver	0.20	0.0305
Newfoundland	0.26	0.1018

^1^Spearman’s rank correlation test. BSD, Belgian Shepherd Dog; CKCS, Cavalier King Charles Spaniel; GSD, German Shepherd Dog.

CRP was the only variable that did not succeed the tests of normal distribution of residuals. Therefore, it was assessed with nonparametric methods and no associations were found with either BCS or with TG.

## Discussion

There are two key findings from this large canine cohort study: first, insulin and cholesterol concentrations were increased in non-obese, overweight dogs [as defined by Brooks et al. (21)] as compared to their lean counterparts; second, when analysing both lean and overweight dogs as a whole, fasting TG concentrations were positively associated with concentrations of cholesterol, glucose, fructosamine and insulin, but negatively associated with FFA. To the best of our knowledge, the current study is the first to report similar changes in a large group of non-obese dogs [i.e., BCS lower than 8 (21)]. The present study is different to previous ones in that the overweight category includes only BCS 6 and 7 (mean 6.1), which are considered to have, respectively, an excess of body fat of 5% (BCS 6) and 10% (BCS 7) (21, 49). Although previous studies have reported increased insulin, cholesterol and TG concentrations in overweight dogs, the “overweight” group in those studies invariably included either some or all dogs in obese body condition [i.e., higher than 7 (21)] (15, 22–25, 29, 33, 50–52). Further, other studies have included dogs with BCS 6/9 within an “ideal weight” group (42, 53, 54). However, dogs with BCS 6 are known to be at greater risk of developing comorbidities, suffering from a poorer quality of life and having a shorter life-expectancy (8–11). Given that there is no consensus about the amount of fat accumulation necessary to trigger pathological consequences in dogs, it seems important to better characterise the physiopathological changes that occur in dogs that are in overweight, but not obese, body condition. Because there are no available studies that focus specifically in the overweight category, and since the biochemical changes in overweight dogs were hypothesised to be similar to those in dogs with obesity, the discussion of the present study largely relies on studies involving obese dogs.

In addition to the main effect of BCS, both TG and cholesterol concentrations were affected by an interaction between overweight status and breed. When such an interaction between two independent variables is found, interpretation of the main effects alone may be misleading. In such instances, each category (in this case, individual breeds) should be investigated independently. Compared with their lean counterparts, overweight dogs of the CKCS breed had greater TG concentrations, whilst overweight dogs of both the Boxer and in the CKCS breeds also had greater cholesterol concentrations.

Although hyperinsulinemia is important in obesity-related disorders in humans (55) and hyperinsulinemia is also a feature of canine obesity (23, 30, 33, 56–60), dogs do not develop the same clinical consequences as humans with MS (15), suggesting that significant physiological and pathophysiological differences might exist between these species (4). In the current study, non-obese overweight status in dogs was associated with increased concentrations of insulin and cholesterol, which might be interpreted as early evidence of ORMD. These changes, although mild and likely not clinically relevant in the short term, might contribute to the long-term consequences of fat accumulation (i.e., reduced lifespan and quality of life, rapid onset of comorbidities) that have been described in both overweight and obese dogs (8–11).

In contrast to TG and cholesterol concentrations, FFA concentrations did not differ between dogs in the overweight and lean categories. Previous studies have shown that both humans and dogs with obesity have increased FFA concentrations, possibly because their concurrent insulin resistance leads to a lack of insulin-mediated suppression of lipolysis (23, 24, 51, 55); further, FFA are considered to be key mediators in the pathogenesis of obesity-induced insulin resistance (55). Therefore, the degree of adiposity in the dogs from the current cohort might have been less than that required to affect circulating FFA concentrations.

In the current study, there was also no difference in glucose concentrations between the dogs in the overweight and lean categories, a finding that contrasts with some (26–28, 61), but not all (15, 22, 24, 29–31, 34, 62) previous studies where glucose concentrations are associated with obesity, weight gain, and weight loss. Fructosamine concentrations were also not different between overweight and lean dogs, again contrasting with previous research where greater fructosamine concentrations were seen in insulin-resistant, but not insulin-sensitive, dogs with obesity (37). The influence of obesity on fructosamine concentrations in humans is believed to be mild (38, 39).

After correcting for the effect of breed, there were positive associations between TG concentrations and the concentrations of glucose, fructosamine, insulin and cholesterol, and also a negative association with FFA concentrations. Some human studies have suggested that TG concentrations are a more sensitive indicator of the risk of developing type 2 DM and cardiovascular complications than having MS, not least in normal-weight humans (63, 64). Similarly, veterinary researchers found that obese dogs with ORMD (as defined by having abnormal values of two of the following: TG, total cholesterol, systolic blood pressure and fasting plasma glucose) had greater insulin and lower adiponectin, without significant differences of body fat. This suggests that biochemical assessment could be helpful classifying dogs at risk of comorbidities irrespective of their level of adiposity (15). Of course, the findings of the present study should be interpreted in light of the fact that none of the dogs were in obese body condition (BCS 8–9). To the author’s knowledge, an association between TG and glucose and fructosamine has not been previously reported in dogs and might reflect an association between increased glucose concentration and altered lipid metabolism. It is important to emphasise that none of the dogs in the present study were considered to have ORMD. Although little research on fructosamine has been undertaken in human MS, this metabolite has been linked to cardiovascular outcomes, increases with dyslipidemia, and is also associated with TG concentrations (65).

Another important finding from this study was the association between fasting TG and insulin concentrations, which has not previously been described in healthy dogs. Increased TG concentrations are commonly associated with both insulin resistance and type 2 DM in humans, and are a central feature of the dyslipidemia that occurs (66–68). In dogs, the development of hypertriglyceridemia is promoted by an increased supply of substrates to the liver, especially glucose and FFA, in insulin-resistant states (23). Previous studies have hypothesised that TG might play a role in compensatory hyperinsulinemia, and that a lack of TG could cause a decrease in insulin compensation for hyperglycaemia (33, 69). This will have an effect on the variability of insulin responses in different physiological states, which should be considered in future studies.

FFA concentrations tend to be greater in insulin-resistant humans because insulin resistance decreases insulin-mediated inhibition of lipolysis, with the effect of increasing FFA concentrations (55). Fatty acids are also mechanistically involved in the pathophysiology of obesity-induced insulin resistance (55). In canine studies, FFA concentrations increase and contribute to insulin resistance (23, 24, 51, 66). In the current study, FFA concentrations were negatively associated with TG concentrations, an unexpected finding given the positive associations seen between TG concentrations and the concentrations of insulin, glucose and fructosamine concentrations, contrasting with previous findings in dogs with ORMD in which all these metabolic markers are increased (55). One possible explanation is that affected dogs in this cohort only had either mild insulin resistance, or were at an early stage, for example, where the liver was affected but not peripheral tissues, previously described (70); in such a state, it could be argued that insulin is unable to inhibit hepatic glucose production whilst still being able to inhibit lipolysis in the periphery. An alternative explanation for the decreased FFA concentration, such as enhanced FFA oxidation, seems unlikely given the other changes identified (e.g., increased insulin, glucose, and fructosamine concentrations).

The acute phase protein CRP is a marker of inflammation and is increased in humans with obesity (41), suggesting possible subclinical inflammation. However, findings have been inconsistent in canine obesity, where an increased CRP concentration has been observed in some (30, 42), but not other (22, 28, 43, 71) studies of canine obesity, including one study where the CRP concentration was less in dogs with obesity (44). In light of this, it is perhaps not surprising that CRP concentrations did not differ between dogs in overweight and lean condition in this study.

As with any study, several limitations should be acknowledged. First, given that only a limited number of breeds were studied, there might be a risk of over-interpreting the breed effects. However, the linear models used were corrected for the effect of the breed. Further, some effects were evident even in overweight dogs within single breeds. Second, the influence of the sex could not be assessed because, apart from Labrador retrievers, the study had been designed in a way that sex was covariate with breed. However, by correcting for the breed effect in linear models, any sex effect would also be corrected, albeit partially. A third limitation was that BCS is an imperfect measure of body fat mass, and is influenced by many factors, including differences in breed morphologies and fat distribution (19), as well as subjectivity when assigning a score. However, all cases were assessed by highly-trained veterinarians, where better agreement would be expected (72). Further, the same investigator was responsible for BCS assessment in each one of the five centres, thus minimising subjectivity.

Also related to BCS, a score of 6 was chosen as a cut-off to divide lean dogs from dogs in overweight because this limit is widely accepted in every-day practise (8, 17–19, 21). When assigning dogs to the overweight or lean categories, there was a predominance of dogs in the lean category in most breeds (≥80% in most breeds, except for CKCS, NF and LAB, which approached 50% in each category). Given such an imbalance, where numbers were small, statistically significant differences between lean and overweight dogs within specific breeds might have been missed. This limitation further shows the interest of using TG concentration as a marker, as the latter can be accurately measured. Indeed, this might have ensured a greater statistical power, explaining the increased number of significant associations with the metabolic variables assessed.

Finally, it could be argued that diet should have been included as a variable in the analyses, for example, by ensuring dogs were fed a limited number of diets. Whilst we acknowledge this limitation, the current study was designed not to test for this parameter and, arguably, assessing a cohort fed a range of different diets is more representative of the situation in client-owned dogs. Therefore, no restriction was imposed in terms of diet, as long as the dogs had only access to a commercial dry food diet and received no supplement during the 3 weeks preceding the analyses.

In conclusion, both BCS and serum TG concentrations were independently associated with changes in markers of lipid and glucose metabolism in this large cohort of healthy, non-obese dogs in lean and overweight condition. This suggests that both BCS and fasting TG concentration may be useful markers of metabolic changes in non-obese dogs. Further analyses using more complex multivariate models are needed to characterise better the interplay between these biochemical analytes.

## Data Availability

The raw data supporting the conclusions of this article will be made available by the authors, without undue reservation.
